# In This Issue

**DOI:** 10.1002/cfg.491

**Published:** 2005


**Comparative and Functional Genomics**
*Comp Funct Genom* 2005; **6**: 267.
Published online in Wiley InterScience (www.interscience.wiley.com). **DOI:** 10.1002/cfg.491

### In this issue

### ARNT transcription read-through into CTSK

The aryl hydrocarbon receptor nuclear translocator (ARNT) and cathepsin K (CTSK) genes lie in a tandem head-to-tail arrangement in extremely close proximity on human chromosome 1. **Fabienne Giraudeau** *et al.* have shown by RT-PCR that ARNT transcripts can extend through the ARNT-CTSK intergenic region into the CTSK gene.

### Comparative analysis of peptidyl-prolyl cis/trans isomerase repertoires

Peptidyl-prolyl cis/trans isomerases are found throughout nature and in all the major compartments of the cell. **Trevor Pemberton and John Kay** have compared the peptidyl-prolyl cis/trans isomerase repertoire of humans to those of the major model organisms in use today to identify the best systems within which to study this family of proteins.

### Characterisation of ESTs from chicken pineal gland

The pineal gland is the circadian oscillator in the chicken, regulating diverse functions ranging from egg laying to feeding. Here, **Stefanie Hartman and coworkers** describe the isolation and characterization of expressed sequence tags (ESTs) from a chicken pineal gland cDNA library. A total of 192 unique sequences were analyzed and submitted to GenBank.

### Conference Report — Sustainability and Governance of Web and GRID Resources in Functional Genomics

**Paul van der Vet** *et al.* report from this ESF Workshop, held at the Fraunhofer Institute at Schloss Birlinghoven, Sankt Augustin, Germany, in May 2005. The meeting aimed to tackle issues relating to efficient interoperability between resources; the need for business models and securing ongoing funding; and the necessity of quality assurance and guidelines.

### Meeting Review — Chicken genomics and development workshop

**Ed Smith and colleagues** report from the Cold Spring Harbor Laboratory Chicken Genomics and Development Workshop held on May 8–11, 2005. The aims of the meeting were to celebrate the recently released draft sequence, compare notes, review progress, and plan for the road ahead. Topics included SNPs, sequence to function, QTL identification, and expression profiling.

### Book Review — Bioinformatics and Molecular Evolution

**Damian Counsell** reviews this textbook by Paul Higgs and Teresa Attwood, finding it to be well-organised and full of sensible practical advice.

### Book Review — RNA Interference in Practice

Alan Lau reviews this practical guide to RNAi from Ute Schepers, discovering it to be an excellent source of reference information for both experienced and novice users.

### Conference Calendar

A listing of genomics related conferences planned for January to March 2006.

Copyright © 2005 John Wiley & Sons, Ltd.

